# Comparing Formation or Non-Formation of Bladder Flap at Cesarean Section on Perioperative and Postoperative Complications: Double-Blind Clinical Trial

**Published:** 2017-09

**Authors:** Farideh Akhlaghi, Azadeh Khazaie, Fateme Jafaripour

**Affiliations:** Department of Obstetrics and Gynecology, Women’s Health Research Center, Mashhad University of Medical Sciences, Mashhad, Iran

**Keywords:** Bladder Flap, Cesarean Section, Bladder

## Abstract

**Objective:** To investigating formation or non-formation of bladder flap at Cesarean section on the complications during and after surgery.

**Materials and methods:** This is a double-blind clinical trial study conducted during February 2014 to May 2015 on 64 pregnant women with gestational age of 36 weeks or more who were delivered by Cesarean section for the first time. They were randomly divided into two groups (intervention group: non-formation of bladder flap; control group: formation of bladder flap). The time to cut out the baby by Cesarean section, total duration of operation, bladder injury, intraoperative bleeding, hematocrit changes expected prior to during and following operation, postoperative pain, macroscopic and microscopic hematuria, postoperative complications and duration of hospitalization were compared between two groups. The data were analyzed with SPSS version 16 using and statistics tests. p < 0.05 was considered significant.

**Results:** Time to cut out the baby for the intervention group (124.9 ± 40.5 seconds and for control group 155.1 ± 42.9 seconds) and total duration of the operation (intervention group: 27.7 ± 5.2 min and control group: 34 ± 4.73 min) were significantly different (p = 0.000). Number of gauze consumption during operation and postoperative hematocrit drop in the intervention group was significantly lower in the intervention group compared the control group (p = 0.000). The postoperative pain score in the intervention group (4.8 ± 1.1) and in control group (6.3 ± 0.9) were significantly different (p = 0.000).

**Conclusion:** Omission of the bladder flap at Cesarean section leads to short-term benefits such as reducing the time to cut out the fetus, duration of surgery, decreasing postoperative bleeding and lowering pain.

## Introduction

Cesarean Section (CS) is one of the most common surgical procedures in the world. In some countries, the cesarean section rates are more than 50% of all deliveries. From 1970 to 2007, cesarean section rates in the United States were recorded from 4.5% of all births to 38%. Efforts to improve cesarean techniques have a long history to reduce the morbidity and mortality resulting from this surgery. So far, several surgical techniques have been reported for CS, which is difference in the type of abdominal incision, formation or non-formation of bladder flap at CS and uterine incision are examples of the various technical differences. Formation of a bladder flap is one of the standard stages in CS. Bladder flap is created through the cutting surface of the visceral peritoneum in order to isolate the bladder from the lower uterine segment. Basically, bladder flap is created for more access to the lower uterine segment in addition to reducing the risk of damage to the bladder during CS (1, 2). However, so far complications such as accumulation of fluid in intraperitoneal space, bladder flap hematoma or bladder flap abscess, urinary retention due to nerve damage, adhesion in the lower uterine segment are created following the formation of a bladder flap at CS (3, 4, 5). Due to these complications, most recently, some experts have been doubtful about the need for routine bladder flap at the time of cesarean delivery. Also, in a meta-analysis published in 2014, it was demonstrated that the formation of a bladder flap will not lead to significantly improved outcomes of CS, and on the other hand, it can prolong the delivery time and, therefore, non-formation of the bladder flap is more advisable (6). However, this study has also noted that the number of intervention studies conducted for comparing the two techniques of formation or non-formation of bladder flap at CS is low and more research should be carried out in this area. This study aims at comparing the two method of formation and none formation of bladder flap during CS delivery on complications during and after surgery.

## Materials and methods

This is a double-blind clinical trial study conducted during February 2014 to May 2015 on pregnant women undergone CS in university hospitals (Imam Reza, and Omolbanin) of Mashhad, Iran. This research have been approved by research ethics committee with code IR.MUMS.REC.1393.133. In this study pregnant women with a gestational age of least 36 weeks that had undergone CS for the first time were enrolled for participation. Exclusion criterias include patients with a history of previous abdominal surgery, with a full dilatation at CS delivery, and zero and lower station. Patients were randomly divided into two groups (intervention group: non-formation of bladder flap and control group: formation of bladder flap). All CS and bladder flap procedures were performed by one surgeon. According to O'Neill et al. study (6), with taking into account alpha and beta as 5% and 20% respectively, the sample size based on the α = 5.00% and β = 20.00%was calculated 64 people for each group. This study was a double-blind clinical trial study so that the participants and researcher were not informed about the process of the intervention and only surgeon was aware of the participant’s groups according to the intervention strategies packet. Upon completion of data collection, each participant’s packet which was representing the created intervention was opened. 

In this study, the participant’s age and gestational age were recorded prior to surgery. Then the length of surgery (from incision to birth and until the end of the skin suturing), blood loss during surgery and estimating blood amount considering the consumed blood gases and the incidence of macroscopic hematuria of surgery were recorded in both groups. Moreover, the occurrence of any unwanted complications during surgery was recorded. After the operation, the hemoglobin drop within 48 hours after surgery compared with before surgery, the microscopic incidence of hematuria at postoperative hospitalization on the basis of urine dipstick (Negative, Trace, 1+, 2+, 3+), the pain level according to postoperative VAS scores (0-10), the need for analgesic drugs during hospitalization, presence or absence of bladder injury during hospitalization, and duration of hospital stays were recorded and all variables were compared between the two group.

Bladder flap formation during CS: This intervention is a part of the standard CS created through the cutting surface of the visceral peritoneum to isolate the bladder from the lower uterine segment (7). 

Avoiding bladder flap formation in CS: to open the bladder flap, a layer of the peritoneum above the upper edge of the bladder and in the anterior part of the lower uterine segment was taken with forceps in the middle line and transected with the scalpel or scissors. The scissors were placed between the bladder – uterine serous and the lower segment myometrium, and pressed out from the center line, and then while the blades were alternately partially opened, it was withdrawn to isolate a strip up to 2 cm wide from the serous, then the incision was made. As we approach the side margins on each side, the scissors were directed in part to the series. The lower flap of the peritoneum was elevated, and the bladder was slowly separated from the underlying myometrium by sharp or slow dissection (8). 

This study was approved by the Ethics Committee of Mashhad University of Medical Sciences (code: IR.MUMS.REC.1393.133), and all patients were informed about the project's objectives before inclusion and signed consent were received from them. 

Participants' privacy and dignity have been observed in the project, and the their coded information were entered to into the Statistical Analysis Program, and the results were published as conclusion 

The collected data were given to SPSS by a computer user. Statistical analysis was performed using SPSS version 19. At first, the situation of data distribution was tested, and Independent t Test was used for parametric data, and Mann-Whitney U Test was used for non-parametric data. Moreover, the chi-square test was applied to compare qualitative data between the two groups. A 95% confidence interval and p < 0.05 were considered as significant level.

## Results

**Figure 1:**
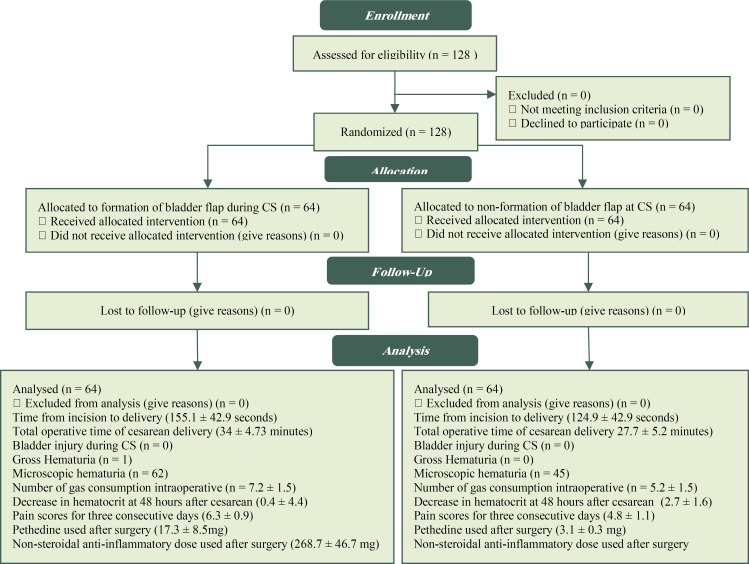
Consort Diagram

**Figure 2:**
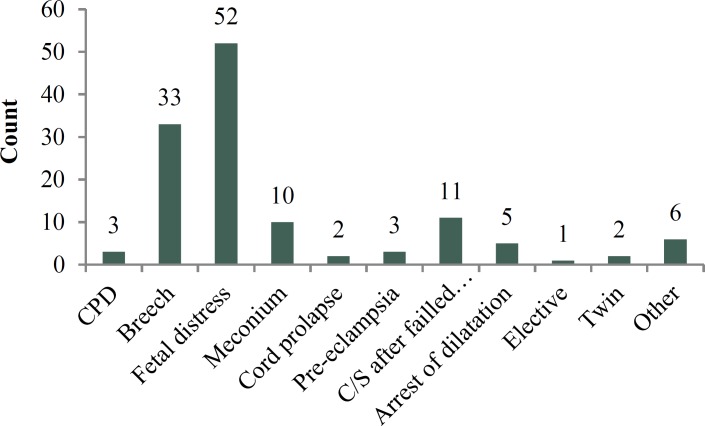
Indication for Cesarean section in all of participants in formation or Non-formation of bladder flap at cesarean section groups

This study included 128 pregnant women who were delivered by CS for the first time. The mean age of them was 26.9 ± 5.9 years and 70% of them was prime gravid, and 30% had experienced of normal vaginal delivery. The mean gestational age was 39.4 ± 1.25 weeks, according to the first day of the last period, and 39.2 ± 1.11 weeks based on ultrasound results. Patients divided to two groups, with the formation of bladder flap during CS (n = 64) and non-formation of bladder flap at CS (n = 64). Mean maternal age in formation of bladder flap group was 26.8 ± 6.0 years and in none formation of bladder flap group was 26.9 ± 6.0 years. (p = 0.844). The mean gestational age in formation of bladder flap group was 39.2 ± 1.1 and in none formation of bladder flap group was 39.7 ± 1.3 (p = 0.381).Two group was similar for age and gestational age and had not differences (Figure 1). The indication for Cesarean section in all of participants was shown in figure 2.

**Table 1:** Comparison of postoperative outcome between the two groups of cesarean section (CS) delivery with and without bladder flap formation

**Variable**	**Unite**	**Bladder flap at** **(CS)** **(N = 64)**	**Omission of** **Bladder flap at CS** **(N = 64)**	**P value0.9**
Microscopic hematuria	Negative	2	19	p = 0.001
	Trace	0	24	
	+1	6	17	
	+2	15	3	
	+3	41	1	
Decrease in hematocrit (48 hours after surgery): percent	Mean ± standard deviation	0.4 ± 4.4	2.7 ± 1.6	p = 0.006
Pain intensity, three times in three days	Mean ± standard deviation	6.3 ± 0.9	4.8 ± 1.1	p = 0.001
Dose of pethidine: mg	Mean ± standard deviation	17.3 ± 8.5	3.1 ± 0.3	p = 0.001
Nonsteroidal anti- inflammatory dosage: mg	Mean ± standard deviation	268.7 ± 46.7	225 ± 43.6	p = 0.001
Complications (Atonic uterus/ Bleeding): number	Number	4	1	p = 0.365
Duration of hospital stay: days	Mean ± standard deviation	1.3 ± 0.5	1.2 ± 0.5	p = 0.001

In the group with bladder flap, the time from incision to delivery was 155.1 ± 42.9 seconds and in the group without bladder flap formation was 124.9 ± 42.9 seconds (Table 1). Differences between two groups was significant (p = 0.000). The total operative time of cesarean delivery in group with bladder flap was 34 ± 4.73 minutes and in group without bladder flap formation was 27.7 ± 5.2 (Table 1). Differences between total operative time in the group without bladder flap formation was significantly lower than the group with the bladder flap formation (p = 0.000). Bladder injury during CS was not observed in any of the samples of the two groups. Also, only in one patient undergone CS with bladder flap formation, Gross Hematuria was seen but differences between two group was not statistically significant (p = 0.801). Microscopic hematuria in two group was seen and the differences between two group was significant (p = 0.001) (Table 2).

In this study the number of gas consumption intraoperative in the bladder flap formation group was 7.2 ± 1.5 and in the none formation bladder flap group was 5.2 ± 1.5 and differences between the two groups was significant (p = 0.000).

**Table 2:** Comparison of operative time between the two groups of cesarean section (CS) delivery with and without bladder flap formation

**Variable**	**Unite**	**Bladder flap at ** **(CS)** **(n = 64)**	**Omission of ** **Bladder flap at CS** **(n = 64)**	**p value**
The time from incision to delivery	Second	155.2	124.98	0.000
Total operative time of cesarean delivery	Minute	34.3	27.75	0.000

**Table 3:** Comparison of postoperative pain and analgesic drugs used after surgery between the two groups of cesarean section (CS) delivery with and without bladder flap formation

**Variable**	**Bladder flap at ** **(CS)**	**Omission of Bladder ** **flap at CS**
Mean of pain scores for three consecutive days after caesarean	6.39	4.87
Mean of pethidine used after surgery	8.59	4.87
Mean of non-steroidal anti-inflammatory dose used after surgery (mg)	268.75	225

The two groups in terms of decrease in hematocrit at 48 hours after cesarean compared to preoperative conditions (Table 2), the pain scores for three consecutive days, pethidine dose and non-steroidal anti-inflammatory dose used after surgery (Table 3) and the number of days of hospitalization (Table 2) had statistically significant differences. But in terms of complications after cesarean, including atonic uterine and bleeding after cesarean, no significant difference was observed between the two groups (Table 2).

## Discussion

This study aims to investigating formation or non-formation of bladder flap at CS on complications during and after surgery. Traditionally, the formation of a bladder flap is one of the most common stages in CS, but recent clinical studies have questioned the necessity and value of this stage. Pelosi and Ortega introduced the first technique for CS by the omission of the bladder flap (9). The theory of omission of the bladder flap in CS delivery has a long history, and the majority of previous studies have examined the effects of this action in short intervals. Therefore, more studies are needed to evaluate the long period of time as well as multiple pregnancies in order to investigate the adhesions or fertility. But some researchers such as Mahajan & Chigbu have mentioned benefits to removing the bladder flap in long-term studies (10, 11). Some of the important factors in the success of CS are the time from skin incision to delivery of the fetus and with less importance, the total operative time at cesarean delivery. Based on the results of this study, the time required for skin incision and removal of fetus and the total operative time at cesarean delivery in the case group (no flap bladder) were significantly less than the control group (no flap bladder). The results of Hohlagschwandtner’s study in this regard are in line with that of the present study (12). Tuuli has pointed out to the time required for skin incision and removal of a fetus affected by omission of the bladder flap; however, the total time of CS has not reduced significantly in this study (13). It has been stated in a study that the pregnant women who eat in the less postoperative period, reduced operative time leads to a reduction in the symptoms of ileus (14). By analyzing the results of studies, and the present study and the meta-analysis by O'Neill et al., it is appeared that the non-formation of bladder flap significantly reduces the time between skin incision and removal of fetus (6). 

Increasing the amount of bleeding during CS is one of the cases that enable a person for involvement with postoperative complications (15). In this study, to estimate the amount of bleeding was performed through the registration of gas consumption to suction blood during surgery. As a result, in the group without a bladder flap, the total number of gas consumption and the drop in hematocrit was significantly lower than another group that could represent a direct impact of omission of the bladder flap on the amount of bleeding during surgery. However, the findings of this study were not consistent with findings of other studies so that in the meta-analyzes conducted on the results of 3 other clinical trial studies, the effect of the flap has not been determined significantly on the bleeding (6). Reducing postoperative pain and the subsequent reduction in the need for analgesics (pethidine and diclofenac in this study) may be due to reduced trauma to the patient during surgery. In this study, in the case group, severity of postoperative pain, pethidine and diclofenac doses compared with the control group significantly decreased. Hohlagschwandtner achieved similar results too. 

Damage to the bladder is one of the rare but fatal complications of CS which was not observed in this study. In this regard, Hohlagschwandtner and Wood pointed to the lack of damage to the bladder similarly (12, 16). Importantly, in conjunction with bladder injury during CS is the rarity of this complication. Some studies have reported a prevalence rate of between 0.14 to 0.31% and pursuant to that proposition, a study with a sample size of over 40,000 is needed for a comprehensive and attributable review around bladder injury during cesarean delivery (2, 17). However, it should be noted that when the lower uterine segment is the thin or fetal head is lower than normal, non-formation of bladder flap can lead to bladder rupture (18). It should be noted in previous studies, two-thirds of damages to the bladder have been reported in women who have had a previous CS and those subjected to emergency CS are most at risk of damage to the bladder during CS (19). 

To check for blood in the urine using urinalysis (U/A), the case group compared with the control group had significantly lower blood and urine. This is probably due to the reduced intraoperative manipulation of the bladder at CS subsequently after the omission of the bladder flap. Hohlagschwandtner has also provided similar results in this respect (12). Gross Hematuria was reported only in a sample through observation of blood in catheter and urine bag. In this study, postoperative complications include atonic uterus and bleeding after CS in 4 cases of the control group and one case of the case group. Hohlagschwandtner has not reported any complications (12). The case group members of this study had significantly shorter postoperative hospitalization stay compared with the control group. However, this variable is influenced by various factors such as previous medical history. In this study, 13 subjects of the case group and 7 subjects of the control group had experienced the previous diseases such as chronic hypertension, pre-eclampsia or BMI (Body Mass Index). In other words, examples of the case group despite having more medical positive records in their samples, but experienced shorter postoperative hospitalization stay. 

The study was faced with various constraints. Samples were not matched for BMI and other anthropometric indices and in this case, the possible role of these indices was not removed on the total operative time at cesarean delivery and the time required for skin incision and removal of the fetus. Also, according to the surgeon’s information to the purpose and grouping of the study, possible differences in the accuracy and speed of surgery in both groups were found. The number of days of hospitalization may be influenced by many factors other than formation of bladder flaps such as post-operative cares. It is recommended, other studies examining the studies variables with long-term follow-up, similar studies in women with previous CS, and women with premature infants to be carried out in future.

## Conclusion

According to results of this study, the omission of the bladder flap at CS is associated with numerous benefits in the short-term study. The benefits include reduction of operating time and incision-delivery interval, reducing the severity of intraoperative bleeding and postoperative hematocrit reduction, pain relief, and analgesic doses and ultimately reducing the postoperative hospitalization stay.
